# Prediction of Methotrexate Clinical Response in Portuguese Rheumatoid Arthritis Patients: Implication of *MTHFR* rs1801133 and *ATIC* rs4673993 Polymorphisms

**DOI:** 10.1155/2014/368681

**Published:** 2014-05-21

**Authors:** Aurea Lima, Joaquim Monteiro, Miguel Bernardes, Hugo Sousa, Rita Azevedo, Vitor Seabra, Rui Medeiros

**Affiliations:** ^1^CESPU, Institute of Research and Advanced Training in Health Sciences and Technologies, Department of Pharmaceutical Sciences, Higher Institute of Health Sciences (ISCS-N), Rua Central de Gandra 1317, 4585-116 Gandra PRD, Portugal; ^2^Molecular Oncology Group CI, Portuguese Institute of Oncology of Porto (IPO-Porto), Rua Dr. António Bernardino de Almeida, 4200-072 Porto, Portugal; ^3^Abel Salazar Institute for the Biomedical Sciences (ICBAS), University of Porto, Rua de Jorge Viterbo Ferreira 228, 4050-313 Porto, Portugal; ^4^Faculty of Medicine of University of Porto (FMUP), Al. Prof. Hernâni Monteiro, 4200-319 Porto, Portugal; ^5^Rheumatology Department, São João Hospital Center, Al. Prof. Hernâni Monteiro, 4200-319 Porto, Portugal; ^6^Virology Service, Portuguese Institute of Oncology of Porto (IPO-Porto), Rua Dr. António Bernardino de Almeida, 4200-072 Porto, Portugal; ^7^Research Department-Portuguese League Against Cancer (LPCC-NRNorte), Estrada Interior da Circunvalação 6657, 4200-177 Porto, Portugal

## Abstract

*Objective.* Methotrexate (MTX), the most used drug in rheumatoid arthritis (RA) treatment, showing variability in clinical response, is often associated with genetic polymorphisms. This study aimed to elucidate the role of methylenetetrahydrofolate reductase (*MTHFR*) C677T and aminoimidazole carboxamide adenosine ribonucleotide transformylase (*ATIC*) T675C polymorphisms and clinicopathological variables in clinical response to MTX in Portuguese RA patients. *Methods.* Study included 233 RA patients treated with MTX for at least six months. *MTHFR* C677T and *ATIC* T675C polymorphisms were genotyped and clinicopathological variables were collected. Statistical analyses were performed and binary logistic regression method adjusted to possible confounding variables. *Results.* Multivariate analyses demonstrated that *MTHFR* 677TT (OR = 4.63; *P* = 0.013) and *ATIC* 675T carriers (OR = 5.16; *P* = 0.013) were associated with over 4-fold increased risk for nonresponse. For clinicopathological variables, noncurrent smokers (OR = 7.98; *P* = 0.001), patients positive to anti-cyclic citrullinated peptide (OR = 3.53; *P* = 0.004) and antinuclear antibodies (OR = 2.28; *P* = 0.045), with higher health assessment questionnaire score (OR = 2.42; *P* = 0.007), and nonsteroidal anti-inflammatory drug users (OR = 2.77; *P* = 0.018) were also associated with nonresponse. Contrarily, subcutaneous administration route (OR = 0.11; *P* < 0.001) was associated with response. *Conclusion.* Our study suggests that *MTHFR* C677T and *ATIC* T675C genotyping combined with clinicopathological data may help to identify patients whom will not benefit from MTX treatment and, therefore, assist clinicians in personalizing RA treatment.

## 1. Introduction

Rheumatoid arthritis (RA) is a chronic disease characterized by an inflammation of the joints with an autoimmune profile and the most widely used disease modifying antirheumatic drug (DMARD) for RA treatment is methotrexate (MTX) [1]. Despite MTX cost-effectiveness, clinical response to MTX varies widely [2]. The factors that are possibly influencing disease course and therapeutic outcome can be classified into (1) clinicopathological variables, which can be divided into patient-related variables (age, gender, ethnicity, and comorbidities), disease-related variables (duration, activity, disability, and biomarkers), and treatment-related variables (compliance, dose, and previous drugs used) [3–9], and (2) genetic factors, such as genetic polymorphisms implicated in key MTX pathway genes [2, 10–15]. Several studies have been performed in order to evaluate the influence of clinicopathological variables in clinical response to MTX [3, 5, 7, 16, 17]; nevertheless, there is no consensus on which factors can be used as predictors [18]. Pharmacogenomics has raised great interest and, in fact, some studies have attempted to clarify the influence of genetic variations on clinical response to MTX [19].

**Figure 1:**
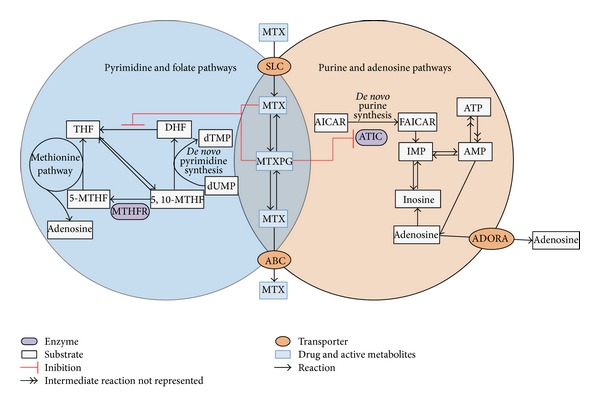
Methotrexate action mechanism. Left panel represents the intervention of MTX in* de novo* pyrimidine synthesis, folate, and methionine pathways by the inhibition of key enzymes. Right panel shows the effect of MTX in* de novo* purine synthesis and adenosine pathway by ATIC inhibition. 5-MTHF: 5-methyltetrahydrofolate; 5,10-MTHF: 5,10-methylenetetrahydrofolate; ABC: ATP-binding cassette; ADORA: adenosine receptor; AICAR: 5-aminoimidazole-4-carboxamide ribonucleotide; AMP: adenosine monophosphate; ATIC: 5-aminoimidazole-4-carboxamide ribonucleotide formyltransferase; ATP: adenosine triphosphate; DHF: dihydrofolate; dTMP: deoxythymidine monophosphate; dUMP: deoxyuridine monophosphate; FAICAR: 5-formamidoimidazole-4-carboxamide ribonucleotide; IMP: inosine monophosphate; MTHFR: methylenetetrahydrofolate reductase; MTX: methotrexate; MTXPG: methotrexate polyglutamate; SLC: solute carrier; THF: tetrahydrofolate.

MTX is an antifolate drug, with antiproliferative and anti-inflammatory effects, by inhibition of folate and adenosine pathways and also inhibition of purines and pyrimidines synthesis (Figure 1) [16, 20, 21]. Methylenetetrahydrofolate reductase (MTHFR), an enzyme involved in folate pathway, is responsible for the conversion of 5,10-methylenetetrahydrofolate (5,10-MTHF) to 5-methyltetrahydrofolate (5-MTHF) that acts as a carbon donor for the remethylation of homocysteine into methionine [22]. On the other hand, methionine can be transformed into S-adenosyl methionine (SAM) and then to S-adenosyl homocysteine (SAH), which can be reversibly hydrolyzed into adenosine and homocysteine [23]. Despite the fact that MTHFR is not directly inhibited by MTX or by its polyglutamated forms (MTXPG), its expression levels seem to influence MTX effect by modifying the folate* status* [16]. Additionally, it is known that aminoimidazole carboxamide adenosine ribonucleotide (AICAR) transformylase (ATIC), an enzyme involved in the* de novo *purine synthesis pathway responsible for the conversion of AICAR into formyl-AICAR (FAICAR), is directly inhibited by MTXPG, causing intracellular accumulation of AICAR [16]. AICAR and its metabolites can then inhibit two enzymes, adenosine deaminase (ADA) and adenosine monophosphate deaminase 1 (AMPD1), which are involved in adenosine metabolism, thus leading to increased intracellular concentrations of adenosine and its consequent release to the extracellular space [21]. This release contributes to the anti-inflammatory effects of MTX since adenosine is a potent anti-inflammatory agent [21].

Several studies have demonstrated that the occurrence of variations on clinical response to MTX could be explained by genetic polymorphisms in* MTHFR* and* ATIC* genes [11, 13–16, 24–28]. The most studied polymorphism in* MTHFR *is C677T (rs1801133), which is responsible for a substitution of an alanine to a valine, leading to a thermolabile form of MTHFR with reduced activity [29]. In fact, it has been suggested that* MTHFR *677T allele is related to MTX nonresponse in RA [13, 24]. Similar to MTHFR, some authors have studied the role of the T675C (rs4673993) polymorphism in* ATIC*, of which the* ATIC* 675C allele has been associated with improved clinical* status* and, consequently, with clinical response to MTX [14, 26].

The pattern of MTX therapeutic outcome is considered to be a major factor for the motivation of researchers and clinicians to enroll patients in pharmacogenetic studies, mainly by comparative studies within different populations. Therefore, the aim of this study was to elucidate the association of clinical response to MTX with* MTHFR* C677T and* ATIC* T675C polymorphisms, in Portuguese RA patients.

## 2. Methods

### 2.1. Characterization of the Studied Population

This study was developed as a retrospective study in a cohort of consecutive Caucasian patients (≥18 years) with RA treated with MTX for at least six months and was conducted between January 2009 and December 2012 at São João Hospital Center (Porto, Portugal). After diagnosis, patients were classified according to the 1987 criteria of the American College of Rheumatology (ACR) and reclassified according to the 2010 criteria of ACR and the European League Against Rheumatism (EULAR) [30]. All patients were initially treated with 10 mg* per os* (PO)/week of MTX in monotherapy. This dose was increased 5 mg at each three weeks if patients did not meet EULAR criteria for response, that is, if presenting a disease activity score in 28 joints (DAS28) > 3.2. At three months, if patients were still without response, the administration route was changed from PO to subcutaneous (SC) maintaining the MTX dose. If within three months, using SC at the maximum tolerable doses, patients did not meet the response criteria, MTX therapy was associated with other synthetic DMARDs. After three more months, if patients continued without response in two successive evaluations and did not present any contraindication, MTX therapy was discontinued or associated with biological DMARDs. The adjustment of MTX therapy also occurred when patients developed MTX-related toxicity. Due to the well-known protective effect of folic acid supplementation for the prevention of toxicity occurrence, in particular for gastrointestinal disorders [31–33], this drug was prescribed once a week to all patients and their regular compliance was registered.

Patients were excluded from the study if not treated with MTX for at least six months and if there was history of drug abuse, recent pregnancy, or desire to become pregnant. The study procedures were considered according to the ethical standards of the Helsinki Declaration by the local Ethical Committee (reference 33/2009) and all patients provided a signed informed written consent.

### 2.2. Data Collection and Variable Definition

Clinicopathological data were collected from individual clinical records by clinicians during patients' regular hospital visits and include variables possibly influencing disease state and clinical response to MTX, which were selected based on either the literature review and/or the clinical significance [3, 5, 7, 16, 17, 33]. These variables included (1) patient-related variables: age, gender, menopause, body mass index (BMI), smoking, number of pack years (NPY), and comorbidities; (2) disease-related variables: diagnosis age, duration, rheumatoid factor (RF), anti-cyclic citrullinated peptide (anti-CCP), antinuclear antibodies (ANAs), DAS28, and health assessment questionnaire (HAQ); and (3) treatment-related variables: symptomatic (corticosteroids and nonsteroidal anti-inflammatory drugs (NSAIDs), supplements (folic acid), other concomitant DMARDs, and MTX administration characteristics (dose, treatment duration, and administration route).

NPY was calculated by the formula: (number of cigarettes smoked per day × number of years smoking)/20. Comorbidity was defined as the presence of diabetes mellitus, hypertension, dyslipidemia, and/or cardiac disorders beyond RA. DAS28 was calculated as described by Prevoo et al. [34]. Daily corticosteroid therapy dose was considered in prednisolone equivalents.

MTX clinical response was recorded at the time of each visit. Nonresponse was defined if patients presented a DAS28 > 3.2 in two consecutive evaluations despite the use of MTX either in monotherapy or combined with other DMARDs. Therefore, at least six months of MTX therapy was required to define which patients had nonresponse to MTX. Response to MTX was defined when patients presented a DAS28 ≤ 3.2.

### 2.3. Sample Collection and Processing

Whole blood samples were obtained with standard venipuncture technique using ethylenediaminetetraacetic acid (EDTA) containing tubes and genomic deoxyribonucleic acid (DNA) extracted with QIAamp DNA Blood Mini Kit according to the manufacturer instructions (QIAGEN, Hilden, Germany). Total genomic DNA was quantified and its purity and integrity were analyzed using the NanoDrop 1000 Spectrophotometer v3.7 (Thermo Scientific, Wilmington, DE, USA).

### 2.4. *MTHFR* C677T and* ATIC* T675C Genotyping

*MTHFR *C677T and* ATIC* T675C polymorphisms were selected based on the role of MTHFR and ATIC in MTX action pathway, upon the putative alteration of these proteins levels and the consequent implication in MTX clinical response [13, 14, 24, 26, 29].

Genotyping protocols were adjusted from those proposed by Sadananda Adiga et al. [35] for* MTHFR* C677T and Hinks et al. [27] for* ATIC* T675C.

*MTHFR* C677T polymorphism was genotyped by polymerase chain reaction-restriction fragment length polymorphism (PCR-RFLP) techniques. PCR amplification was performed for a final volume of 50 *μ*L containing 0.3 *μ*M of each primer (forward: 5′-TGA AGG AGA AGG TGT CTG CGG GA-3′; reverse: 5′-AGG ACG GTG CGG TGA GAG TG-3′), 1x DreamTaq Green master mix (Thermo Scientific, Vilnius, Lithuania), and 50–100 ng of genomic DNA. The PCR conditions consisted of initial denaturation at 94°C during 5 minutes followed by 30 cycles with denaturation for 1 minute at 94°C, annealing for 1 minute at 57°C, extension for 15 seconds at 72°C, and a final extension at 72°C during 10 minutes. RFLP was performed at 37°C, overnight, using HinfI (Thermo Scientific, Vilnius, Lithuania). Individuals with the CC genotype presented 1 fragment with 198 base pairs (bp), whereas individuals with the TT genotype presented 1 fragment with 175 bp.

*ATIC* T675C polymorphism was genotyped using TaqMan SNP Genotyping Assay (C_362264_10) from Applied Biosystems (Foster City, CA, USA) with fluorogenic binding probes. Reactions were performed on an Applied Biosystems 7300 Real Time PCR System (Applied Biosystems, Foster City, CA, USA) with a 5 *μ*L final volume mixture containing 1x TaqMan Genotyping Master Mix (Applied Biosystems, Foster City, CA, USA), 900 nM of each primer, 200 nM of probes labeled with either FAM or VIC, and 10 ng of extracted DNA. Thermal cycling conditions were 10 minutes at 95°C followed by 40 cycles of 15 seconds at 95°C and 1 minute at 60°C. Allelic discrimination was performed by measuring endpoint fluorescence using ABI PRISM Sequence Detection System (Version 1.2.3, Applied Biosystems, Foster City, CA, USA).

For quality control, 10% of the samples were randomly selected for a second analysis and 10% percent of cases were confirmed by automated sequencing in a 3130xl Genetic Analyzer using the Kit BigDye Terminator v3.1 (Life Technologies, Foster City, CA, USA). Results were 100% concordant.

### 2.5. Statistical Analysis

Statistical analyses were performed using the IBM SPSS Statistics for Windows, Version 20.0 (IBM Corp., Armonk, NY, USA), considering a statistically significant probability (*P*) value of 5% or less. The chi-square test was used to assess the association between the groups (response* versus *nonresponse) and the different categorical variables. Odds ratio (OR) and the correspondent 95% confidence intervals (CI) were calculated as a measure of the association between the categorical variables. For the comparison of quantitative variables two sample *t*-tests and nonparametric Mann-Whitney *U* tests were applied.

Multivariate analysis with binary logistic regression was used to identify which genetic variables (*MTHFR *C677T and* ATIC *T675C genotypes) and clinicopathological variables could predict risk for occurrence of nonresponse to MTX. This analysis was performed adjusting to potential confounding variables in three steps: (1) patient-related variables; (2) patient- and disease-related variables; and (3) patient-, disease-, and treatment-related variables.

## 3. Results

### 3.1. Characterization of the Studied Population

Table 1 reports the clinicopathological variables of population enrolled in the study, that includes follow-up data from a total of 233 patients (196 females and 37 males), with a mean age of 52 ± 11.9 and disease duration of 8.0 (range: 0.5–53.0) years. Considering MTX therapy, the median treatment duration was 28.0 (range: 6.0–230.0) months with a median dose of 15.0 (range: 2.5–25.0) mg/week. Furthermore, 201 patients (86.3%) administered MTX by PO administration route and 32 (13.7%) by SC administration route. Nonresponse to MTX was observed in 128 (54.9%) patients and the mean for DAS28 was 4.2 ± 1.3.

### 3.2. Clinicopathological Variables and Clinical Response to MTX

Table 2 represents the relation between clinicopathological variables and clinical response to MTX. In accordance with patient-related variables, our results showed that early age of diagnosis (*P* < 0.001) and noncurrent smokers (OR = 0.32; *P* = 0.004) were statistically significant associated with nonresponse to MTX. Concerning disease-related variables, our results demonstrated that positivity to anti-CCP (OR = 2.28; *P* = 0.007) and ANAs (OR = 1.98; *P* = 0.024) was statistically significant associated with nonresponse to MTX. Additionally, higher number of tender joints count (TJC) (*P* = 0.007) and swollen joints count (SJC) (*P* = 0.008) and higher health assessment questionnaire (HAQ) score (*P* = 0.006) were statistically significant associated with nonresponse to MTX. Considering the treatment-related variables, our results revealed that NSAIDs users (OR = 3.09; *P* < 0.001) were associated with nonresponse to MTX. In addition, attending to MTX administration characteristics, higher MTX doses (*P* < 0.001) were associated with nonresponse to MTX, while SC administration route (OR = 0.32; *P* = 0.004) was statistically significant associated with response to MTX.

### 3.3. *MTHFR* C677T and* ATIC* T675C and Clinical Response to MTX

The frequencies of* MTHFR* C677T (rs1801133) genotypes were 105 CC (45.1%), 99 CT (42.5%), and 29 TT (12.4%), while for* ATIC* T675C (rs4673993) they were 110 TT (47.2%), 99 TC (42.5%), and 24 CC (10.3%). In our population, the minor allele for* MTHFR* C677T was T and for* ATIC* T675C was C (see Figure S1 in Supplementary Materials available online at http://dx.doi.org/10.1155/2014/368681). Considering distribution between responders and nonresponders, results showed significant differences for* MTHFR* C667T (*P* = 0.049) and* ATIC* T675C (*P* = 0.025) genotypes.

Table 3 and Figures S2 and S3 represent the relation between genetic variables and clinical response to MTX. In accordance with* MTHFR* C677T polymorphism, our results showed that* MTHFR* 677TT was statistically significant associated with about 3-fold increased risk for nonresponse to MTX when compared to* MTHFR* 677CC (OR = 3.08; *P* = 0.015) and* MTHFR* 677C carriers (OR = 2.91; *P* = 0.015). Regarding* ATIC* T675C polymorphism, we observed that* ATIC* 675CC was associated with response to MTX when compared to* ATIC* 675TT (OR = 0.32; *P* = 0.016) and* ATIC* 675T carriers (OR = 0.30; *P* = 0.007).

### 3.4. Multivariate Analysis and Clinical Response to MTX

Multivariate analysis with binary logistic regression was used to identify which clinicopathological and genetic variables (*MTHFR* C677T and* ATIC* T675C genotypes) could predict risk for the occurrence of nonresponse to MTX (Table 4). This analysis was performed in three steps adjusting to potential confounding variables. In the first step, patient-related variables were considered and our results demonstrated that* MTHFR* 677TT (OR = 2.64; *P* = 0.040) and* ATIC* 675T carriers (OR = 3.20; *P* = 0.022) were associated with about 3-fold increased risk for nonresponse to MTX. In a second step, beyond patient-related variables, disease-related variables were added and results confirmed that* MTHFR* 677TT (OR = 3.23; *P* = 0.025) and* ATIC* 675T carriers (OR = 4.63; *P* = 0.007) were associated with nonresponse to MTX. In a third step, beyond patient- and disease-related variables, treatment-related variables were added and the obtained results showed that* MTHFR* 677TT carriers (OR = 4.63; *P* = 0.013) were statistically significant associated with more than 4-fold increased risk for nonresponse to MTX when compared to* MTHFR* 677C carriers. Additionally,* ATIC* 675T carriers (OR = 5.16; *P* = 0.013) were statistically significant associated with more than 5-fold increased risk for nonresponse to MTX when compared to* ATIC* 675CC.

Furthermore, considering clinicopathological variables, we observed that noncurrent smokers (OR = 7.98; *P* = 0.001), positivity to anti-CCP (OR = 3.53; *P* = 0.004) and ANAs (OR = 2.28; *P* = 0.045), higher HAQ (OR = 2.42; *P* = 0.007), and NSAIDs users (OR = 2.77; *P* = 0.018) were statistically significant associated with nonresponse to MTX. Moreover, SC administration route (OR = 0.11; *P* < 0.001) was statistically significant associated with response to MTX.

## 4. Discussion

Despite the fact that MTX is extensively used in RA treatment, the individual clinical response to MTX is variable and, therefore, additional DMARDs are often required to achieve a low disease activity profile or even remission [2].

Previous studies revealed controversial results when clinicopathological variables were associated with* MTHFR* C677T and* ATIC* T675C polymorphisms for clinical response to MTX. Several explanations can be proposed for such observed discrepancies, such as bias related to study design and settings, sample size/power, ethnicity, the population disease duration (early or established RA), changes in folate* status*, influence of less common single nucleotide polymorphisms (SNPs) in* MTHFR* and* ATIC*, polymorphisms in genes encoding to other intervenient proteins in folate, purine, pyrimidine, adenosine, and methionine pathways, and also differences in the definition of MTX clinical response [28].

Besides the potential importance of our results, we are aware of possible limitations, especially the sample size. Despite this, patient characteristics are similar to those reported in the literature [36, 37]. Our case series is a representative clinical practice cohort of established and well-defined RA patients [25, 38] and the genotypes distribution of* MTHFR* C677T and* ATIC* T675C polymorphisms is in accordance with the published literature for other Caucasian population [13, 14, 24–26, 39].

### 4.1. *MTHFR* C677T and* ATIC* T675C and Clinical Response to MTX

Regarding* MTHFR *C677T polymorphism, our results demonstrated a statistically significant association between* MTHFR *677TT and nonresponse to MTX, which is in accordance with previously reported studies [13, 24]. Although MTHFR is not directly inhibited by MTX or MTXPG, its expression levels may play an important role in MTX overall effect by modifying the folate* status *of the cell [16]. Literature describes* MTHFR *677TT as responsible for a reduction of MTHFR activity [29], leading to reduced 5-MTHF and other folate cofactors levels and, consequently, to decreased adenosine release [22, 23, 40], which can partially explain MTX nonresponse.

Regarding* ATIC *T675C polymorphism, our results indicate that* ATIC *675T carriers presented an increased risk for nonresponse to MTX, as previously reported [14, 26]. To the best of our knowledge, there are no functional studies reporting the effect of this polymorphism in ATIC activity. Nevertheless, it can be hypothesized that the presence of* ATIC *675T allele will lead to MTX nonresponse due to the increased conversion of AICAR to FAICAR (Figure 1), causing adenosine degradation and its nonrelease, hindering MTX anti-inflammatory effects. Additionally,* ATIC *675T allele seems to contribute to the decrease of MTX antiproliferative effect [41]. Moreover, this polymorphism seems to be in linkage disequilibrium with* ATIC* C347G (rs2372536), of which* ATIC* 347G carriers (minor allele) have been reported as related to better response [16, 26, 42, 43]. Hence, results are consistent with ours reporting an association between* ATIC *675CC (minor allele) and clinical response to MTX.

### 4.2. Clinicopathological Variables and Clinical Response to MTX

According to patient-related variables, multivariate analysis results demonstrated that noncurrent smokers were associated with nonresponse to MTX. Literature describes the association between smoking and decreased folate levels which, in fact, enhance the antifolate effect of MTX and, therefore, improve clinical response to MTX [44–46]. Furthermore, cigarette nicotine seems to potentiate the immunosuppressive and anti-inflammatory effects by acting on the immunological system [47, 48]. Although some studies have demonstrated that smokers had worst response to MTX, presenting a higher disease activity and severity [6, 49], others were able to demonstrate that tobacco exposure reduced radiographic progression and favored a better functional score [50, 51]. Considering disease-related variables, our results demonstrated an association of more than 2-fold higher risk between anti-CCP and ANAs positivity and nonresponse to MTX. Anti-CCP and ANAs are autoantibodies found in RA that are strongly correlated with erosive disease, worse functional* status,* and higher disease activity [1, 9, 52–55] associated with nonresponse. Other studies have shown a relation between anti-CCP positivity and MTX response or presented no associations in early RA patients [56, 57]; nevertheless, our results may be explained by the fact that our series was constituted mainly by patients with established disease. To the best of our knowledge there are no studies in RA associating ANAs and MTX response. Additionally, higher HAQ was associated with more than 2-fold increased risk for nonresponse to MTX. Since higher HAQ score represents an increased disease activity it was expected, as reported by others, that these patients have worst response [56, 58]. In accordance with treatment-related variables, the concomitant use of NSAIDs was correlated with nonresponse to MTX. These results could be explained by the existence of drug-drug interactions since NSAIDs are known to alter MTX and 7-hydroxymethotrexate binding to plasmatic proteins and to impair MTX hepatic metabolism [41]. This translates into low amount of free MTX and lesser formation of active MTX metabolites in hepatocytes. Due to the importance of NSAIDs as symptomatic therapy in RA and due to contradictory results reported, further studies are required to clarify this association [56, 59]. In addition, SC administration route was statistically significant associated with MTX response. This result can be explained by the higher MTX bioavailability associated with SC administration route [60]. Consequently, this will lead to a greater tissues exposure to MTX, higher cellular polyglutamation and retention, and better response to MTX.

## 5. Conclusions

Our results suggested that noncurrent smoking, anti-CCP and ANAs positivity, higher HAQ, NSAIDs utilization, PO administration route, T homozygosity for* MTHFR* C677T, and T allele carrying for* ATIC* T675C can be possible predictive factors of nonresponse to MTX. Thus, the inclusion of these polymorphisms in combination with clinicopathological variables may add valuable information that may help to identify patients who will benefit from MTX treatment and assist clinicians to make better treatment decisions. Despite the potential of these findings, translation into clinical practice requires larger and multicentric studies in order to clearly endorse the importance of these polymorphisms.

## Supplementary Material


Figure S1. Genotype distribution for *MTHFR* C677T and *ATIC* T675C polymorphisms of population enrolled in the study.
*ATIC*: 5-aminoimidazole-4-carboxamide ribonucleotide formyltransferase; C: cytosine; *MTHFR*: methylenetetrahydrofolate reductase; T: thymine.
Figure S2. Relation between *MTHFR* C677T genotypes and clinical response to methotrexate.
*P* value <0.05 is considered to be of statistical significance (highlighted in bold) when compared to reference genotype(s). C: cytosine; *MTHFR*: methylenetetrahydrofolate reductase; T: thymine.
Figure S3. Relation between *MTHFR* T675C genotypes and clinical response to methotrexate.
*P* value <0.05 is considered to be of statistical significance (highlighted in bold) when compared to reference genotype(s). *ATIC*: 5-aminoimidazole-4-carboxamide ribonucleotide formyltransferase; C: cytosine; T: thymine.

## Figures and Tables

**Table 1:** Clinicopathological variables of population enrolled in the study.

	Value
*Patient-related *	
Male, *n* (%)	37 (15.9)
Female, *n* (%)	196 (84.1)
Postmenopausal, *n* (%)	96 (49.0)
Current smokers, *n* (%)	32 (13.7)
NPY*, median (IQR)	19.5 (0.8–120.0)
Comorbidity**, *n* (%)	126 (54.1)

*Disease-related *	
Diagnosis age, mean ± SD, years	40.3 ± 13.2
Disease duration, median (IQR), years	8.0 (0.5–53.0)
RF positive, *n* (%)	131 (56.2)
Anti-CCP positive, *n* (%)	175 (75.1)
ANAs positive, *n* (%)	66 (28.3)
DAS28, mean ± SD	4.2 ± 1.3
Individual variables—DAS28	
TJC (out of 28), median (IQR)	4.0 (0.0–27.0)
SJC (out of 28), median (IQR)	3.0 (0.0–24.1)
ESR, median (IQR), minutes (1st hour)	18.0 (1.0–92.0)
Global health on VAS, median (IQR)	48.0 (0.0–100.0)
HAQ score, median (IQR)	1.25 (0.0–2.9)
HAQ ≤ 0.5, *n* (%)	39 (16.7)

*Treatment-related * ^§^	
Symptomatic	
Corticosteroids, *n* (%)	188 (80.7)
Daily dose in prednisolone equivalents, median (IQR), mg	5.0 (0.0–20.0)
NSAIDs, *n* (%)	170 (73.0)
Supplements	
Folic acid^#^, *n* (%)	118 (50.6)
DMARDs	
Methotrexate monotherapy, *n* (%)	146 (62.7)
Combined methotrexate therapy—synthetic DMARDs, *n* (%)	59 (25.3)
Combined methotrexate therapy—biological DMARDs, *n* (%)	28 (12.0)
Methotrexate administration characteristics	
Dose, median (IQR), mg/week	15.0 (2.5–25.0)
Treatment duration, median (IQR), months	28.0 (6.0–230.0)
*Per os* administration route, *n* (%)	201 (86.3)
Subcutaneous administration route, *n* (%)	32 (13.7)

*NPY = (number of cigarettes smoked per day × number of years smoking)/20.

**Comorbidity was defined as the presence of diabetes mellitus, hypertension, dyslipidemia, and/or cardiac disorders beyond rheumatoid arthritis.

^§^Drugs coadministered with methotrexate when clinical response to methotrexate was recorded.

^#^Patients in compliance with folic acid supplementation.

ANAs: antinuclear antibodies; Anti-CCP: anti-cyclic citrullinated peptide; BMI: body mass index; DAS28: disease activity score 28; DMARDs: disease modifying antirheumatic drugs; ESR: erythrocyte sedimentation rate; HAQ: health assessment questionnaire; IQR: interquartile range; NPY: number of pack years; NSAIDs: nonsteroidal anti-inflammatory drugs; RF: rheumatoid factor; SD: standard deviation; SJC: swollen joints count; TJC: tender joints count; VAS: visual analog scale.

**Table 2:** Relation between clinicopathological variables and clinical response to methotrexate.

Characteristic	Response (*n* = 105)	Nonresponse (*n* = 128)	*P* value
*Patient-related *			
Male, *n* (%)	19 (51.4)	18 (48.6)	Reference
Female, *n* (%)	86 (43.9)	110 (56.1)	0.402
Premenopausal, *n* (%)	39 (39.0)	61 (61.0)	Reference
Postmenopausal, *n* (%)	47 (49.0)	49 (51.0)	0.160
Age, mean ± SD, years	55.1 ± 11.6	49.3 ± 11.5	**<0.001**
BMI, median (IQR), Kg/m^2^	26.2 (18.5–43.1)	26.3 (18.4–38.9)	0.574
Noncurrent smoker*, *n* (%)	83 (41.3)	118 (58.7)	Reference
Current smoker, *n* (%)	22 (68.8)	10 (31.2)	0.004^a^
NPY**, median (IQR)	20.1 (1.5–120.0)	14.0 (0.8–40.0)	0.269
Noncomorbidity, *n* (%)	51 (47.7)	56 (52.3)	Reference
Comorbidity***, *n* (%)	54 (42.9)	72 (57.1)	0.462

*Disease-related *			
Diagnosis age, mean ± SD, years	42.1 ± 13.3	39.1 ± 12.8	0.081
Disease duration, median (IQR), years	8.0 (1.0–53.0)	8.0 (0.5–38.0)	0.164
RF negative, *n* (%)	42 (41.2)	60 (58.8)	Reference
RF positive, *n* (%)	63 (48.1)	68 (51.9)	0.293
Anti-CCP negative, *n* (%)	35 (60.3)	23 (39.7)	Reference
Anti-CCP positive, *n* (%)	70 (40.0)	105 (60.0)	0.007^b^
ANAs negative, *n* (%)	83 (49.7)	84 (50.3)	Reference
ANAs positive, *n* (%)	22 (33.3)	44 (66.7)	0.024^c^
DAS28, mean ± SD	4.0 ± 1.5	4.3 ± 1.2	0.089
Individual variables—DAS28			
TJC (out of 28), median (IQR)	3.0 (0.0–27.0)	5.0 (0.0–20.0)	**0.007**
SJC (out of 28), median (IQR)	2.0 (0.0–24.0)	4.0 (0.0–23.0)	**0.008**
ESR, median (IQR), minutes (1st hour)	19.0 (1.0–88.0)	17.0 (1.0–92.0)	0.509
Global health on VAS, median (IQR)	47.0 (0.0–100.0)	49.0 (0.0–100.0)	0.516
HAQ score, median (IQR)	1.1 (0.0–2.9)	1.5 (0.0–2.6)	**0.006**

*Treatment-related * ^§^			
Symptomatic			
Noncorticosteroids, *n* (%)	21 (46.7)	24 (53.3)	Reference
Corticosteroids, *n* (%)	84 (44.7)	104 (55.3)	0.810
Non-NSAIDs, *n* (%)	41 (65.1)	22 (34.9)	Reference
NSAIDs, *n* (%)	64 (37.6)	106 (62.4)	<0.001^d^
Supplements			
Folic acid nonregular users, *n* (%)	52 (45.2)	63 (54.8)	Reference
Folic acid regular users, *n* (%)	53 (44.9)	65 (55.1)	0.963
Methotrexate administration characteristics			
Dose, median (IQR), mg/week	15.0 (2.5–25.0)	20.0 (7.5–25.0)	**<0.001**
Treatment duration, median (IQR), months	28.0 (6.0–230.0)	29.0 (6.0–209.0)	0.204
*Per os* administration route, *n* (%)	83 (41.3)	118 (58.7)	Reference
Subcutaneous administration route, *n* (%)	22 (68.8)	10 (31.2)	0.004^e^

*Noncurrent smokers include the never smokers and the ex-smokers.

**NPY = (number of cigarettes smoked per day × number of years smoking)/20.

***Comorbidity was defined as the presence of diabetes mellitus, hypertension, dyslipidemia, and/or cardiac disorders beyond rheumatoid arthritis.

^§^Drugs coadministered with methotrexate when clinical response to methotrexate was recorded.

*P* value < 0.05 is considered to be of statistical significance (highlighted in bold).

^a^OR = 0.32, 95% CI: 0.14–0.71. ^b^OR = 2.28, 95% CI: 1.24–4.19. ^c^OR = 1.98, 95% CI: 1.09–3.58. ^d^OR = 3.09, 95% CI: 1.69–5.65. ^e^OR = 0.32, 95% CI: 0.14–0.71.

ANAs: antinuclear antibodies; anti-CCP: anti-cyclic citrullinated peptide; BMI: body mass index; DAS28: disease activity score 28; ESR: erythrocyte sedimentation rate; HAQ: health assessment questionnaire; IQR: interquartile range; NPY: number of pack years; NSAIDs: nonsteroidal anti-inflammatory drugs; RF: rheumatoid factor; SD: standard deviation; SJC: swollen joints count; TJC: tender joints count; VAS: visual analog scale.

**Table 3:** Relation between genetic variables and clinical response to methotrexate.

	Response (*n* = 105)	Nonresponse (*n* = 128)	*P* value	OR (95% CI)
*MTHFR* C677T, rs1801133				
CC	52 (49.5)	53 (50.5)		Reference
CT	46 (46.5)	53 (53.5)	0.662	1.13 (0.65–1.96)
TT	7 (24.1)	22 (75.9)	**0.015**	3.08 (1.21–7.84)
CC	52 (49.5)	53 (50.5)		Reference
T carrier	53 (41.4)	75 (58.6)	0.215	1.39 (0.83–2.33)
C carrier	98 (48.0)	106 (52.0)		Reference
TT	7 (24.1)	22 (75.9)	**0.015**	2.91 (1.19–7.10)

*ATIC* T675C, rs4673993				
TT	48 (43.6)	62 (56.4)		Reference
TC	40 (40.4)	59 (59.6)	0.637	1.14 (0.66–1.98)
CC	17 (70.8)	7 (29.2)	0.016	0.32 (0.12–0.83)
TT	48 (43.6)	62 (56.4)		Reference
C carrier	57 (46.3)	66 (53.7)	0.679	0.90 (0.53–1.50)
T carrier	88 (42.1)	121 (57.9)		Reference
CC	17 (70.8)	7 (29.2)	0.007	0.30 (0.12–0.75)

Results are expressed in *n* (%).

*P* value < 0.05 is considered to be of statistical significance (highlighted in bold).

ATIC: 5-aminoimidazole-4-carboxamide ribonucleotide formyltransferase; C: cytosine; CI: confidence interval; MTHFR: methylenetetrahydrofolate reductase; OR: odds ratio; T: thymine.

**Table 4:** Multivariate logistic regression analysis and clinical response to methotrexate.

Genetic variables	Adjusted variables					
	Patient-related		Patient-related + disease-related		Patient-related + disease-related + treatment-related	
	*P* value	OR (95% CI)	*P* value	OR (95% CI)	*P* value	OR (95% CI)

*MTHFR* C677T, rs1801133						
C carriers		Reference		Reference		Reference
TT	**0.040**	2.64 (1.04–6.67)	**0.025**	3.23 (1.16–9.02)	**0.013**	4.63 (1.37–15.60)

*ATIC* T675C, rs4673993						
CC		Reference		Reference		Reference
T carriers	**0.022**	3.20 (1.18–8.66)	**0.007**	4.63 (1.51–14.12)	**0.013**	5.16 (1.42–18.76)

*P* value < 0.05 is considered to be of statistical significance (highlighted in bold).

Adjusted variables include (1) patient-related variables (age, gender, and smoking), (2) disease-related variables (diagnosis age, disease duration, anti-CCPs, ANAs, TJC, SJC, and HAQ), and (3) treatment-related variables (folic acid supplementation, corticosteroids therapy, use of NSAIDs, other concomitant DMARDs used and MTX administration characteristics such as dose, treatment duration, and administration route). Genetic variables include *MTHFR *C677T and *ATIC* T675C polymorphisms.

ANAs: antinuclear antibodies; anti-CCP: anti-cyclic citrullinated peptide; ATIC: 5-aminoimidazole-4-carboxamide ribonucleotide formyltransferase; C: cytosine; CI: confidence interval; HAQ: health assessment questionnaire; MTHFR: methylenetetrahydrofolate reductase; NSAIDs: nonsteroidal anti-inflammatory drugs; OR: odds ratio; SJC: swollen joints count; T: thymine; TJC: tender joints count.
