# Supplementary material for: Prediction of Methotrexate Clinical Response in Portuguese Rheumatoid Arthritis Patients: Implication of MTHFR rs1801133 and ATIC rs4673993 Polymorphisms
Source: Biomed Res Int. 2014 May 21;2014:368681. doi: 10.1155/2014/368681 (PMC4055378; doi:10.1155/2014/368681)
Supplement: Supplementary file 1 — Figure S1. Genotype distribution for MTHFR C677T and ATIC T675C polymorphisms of population enrolled in the study. ATIC: 5-aminoimidazole-4-carboxamide ribonucleotide formyltransferase; C: cytosine; MTHFR: methylenetetrahydrofolate reductase; T: thymine. Figure S2. Relation between MTHFR C677T genotypes and clinical response to methotrexate. P value <0.05 is considered to be of statistical significance (highlighted in bold) when compared to reference genotype(s). C: cytosine; MTHFR: methylenetetrahydrofolate reductase; T: thymine. Figure S3. Relation between MTHFR T675C genotypes and clinical response to methotrexate. P value <0.05 is considered to be of statistical significance (highlighted in bold) when compared to reference genotype(s). ATIC: 5-aminoimidazole-4-carboxamide ribonucleotide formyltransferase; C: cytosine; T: thymine. [file 368681.f1.pdf]

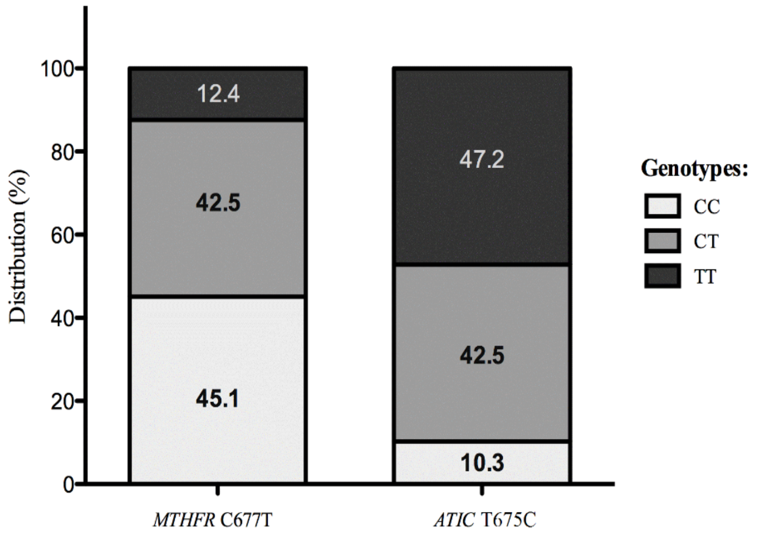

Figure S1. Genotype distribution for *MTHFR* C677T and *ATIC* T675C polymorphisms.

ATIC: 5-aminoimidazole-4-carboxamide ribonucleotide formyltransferase; C: cytosine; MTHFR: methylenetetrahydrofolate reductase; T: thymine.

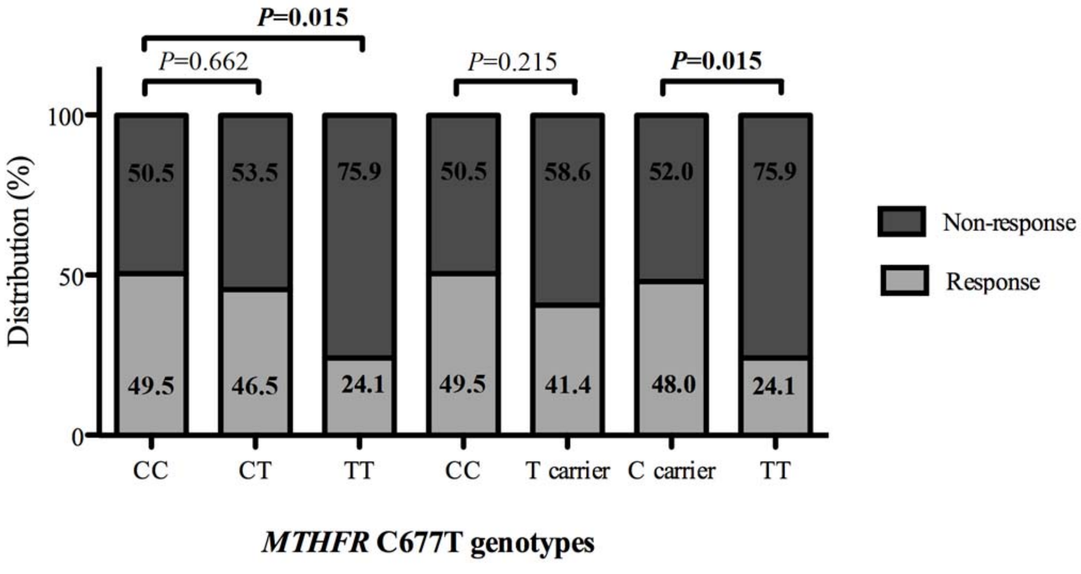

Figure S2. Relation between *MTHFR* C677T genotypes and methotrexate clinical response.

P value <0.05 is considered to be of statistical significance (highlighted in bold) when compared to reference genotype(s).

C: cytosine; MTHFR: methylenetetrahydrofolate reductase; T: thymine.

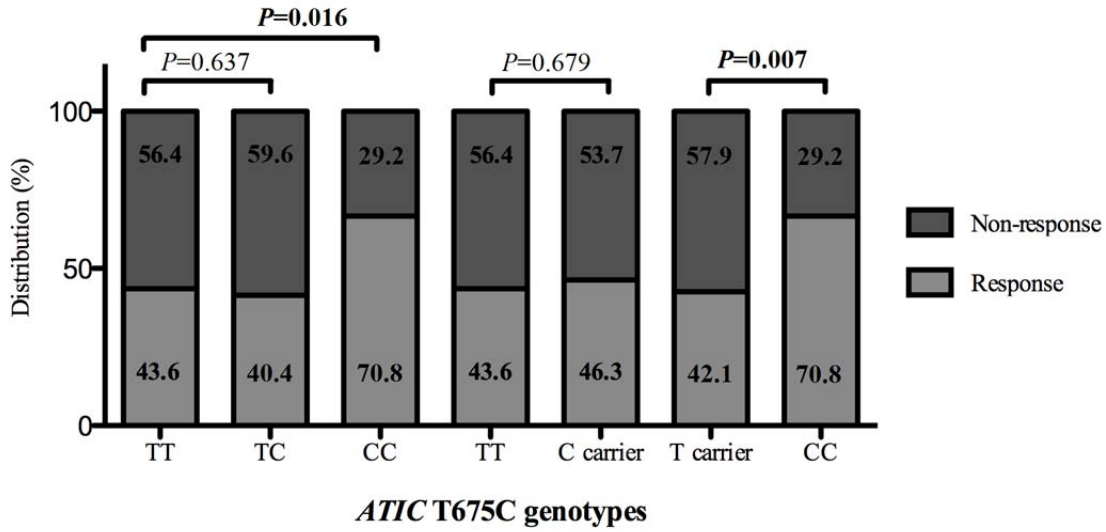

Figure S3. Relation between *ATIC* T675C genotypes and methotrexate clinical response.

P value <0.05 is considered to be of statistical significance (highlighted in bold) when compared to reference genotype(s).

ATIC: 5-aminoimidazole-4-carboxamide ribonucleotide formyltransferase; C: cytosine; T: thymine.
